# Dolphins Can Maintain Vigilant Behavior through Echolocation for 15 Days without Interruption or Cognitive Impairment

**DOI:** 10.1371/journal.pone.0047478

**Published:** 2012-10-17

**Authors:** Brian K. Branstetter, James J. Finneran, Elizabeth A. Fletcher, Brian C. Weisman, Sam H. Ridgway

**Affiliations:** 1 National Marine Mammal Foundation, San Diego, California, United States of America; 2 U.S. Navy Marine Mammal Program, Space and Naval Warfare Systems Center Pacific, San Diego, California, United States of America; 3 Maritime Services Division, Science Applications International Corporation, San Diego, California, United States of America; University of Western Ontario, Canada

## Abstract

In dolphins, natural selection has developed unihemispheric sleep where alternating hemispheres of their brain stay awake. This allows dolphins to maintain consciousness in response to respiratory demands of the ocean. Unihemispheric sleep may also allow dolphins to maintain vigilant states over long periods of time. Because of the relatively poor visibility in the ocean, dolphins use echolocation to interrogate their environment. During echolocation, dolphin produce clicks and listen to returning echoes to determine the location and identity of objects. The extent to which individual dolphins are able to maintain continuous vigilance through this active sense is unknown. Here we show that dolphins may continuously echolocate and accurately report the presence of targets for at least 15 days without interruption. During a total of three sessions, each lasting five days, two dolphins maintained echolocation behaviors while successfully detecting and reporting targets. Overall performance was between 75 to 86% correct for one dolphin and 97 to 99% correct for a second dolphin. Both animals demonstrated diel patterns in echolocation behavior. A 15-day testing session with one dolphin resulted in near perfect performance with no significant decrement over time. Our results demonstrate that dolphins can continuously monitor their environment and maintain long-term vigilant behavior through echolocation.

## Introduction

During echolocation, an animal produces a sound and listens to returning echoes to gain information about its environment. For a few animal species such as bats and odontocetes (e.g., toothed whales such as dolphins and porpoises), echolocation is an important, if not primary means of finding and capturing prey [1], [2], navigating [3], [4], maintaining group cohesion [1] and potentially avoiding predators. Dolphins have evolved to exploit echolocation due to the relative poor visibility of the ocean [5] and efficient sound transmission in water. The echolocation signals of the bottlenose dolphin can be described as a series of broadband transient clicks, with peak frequencies typically between 40 and 120 kHz, durations as small as 40 µsec, with a peak-to-peak sound pressure level (SPL_p-p_) often exceeding 200 dB re 1 µPa [6]. These signals are projected forward in a tight beam [6] where the width and direction of the beam can be actively manipulated depending on target location and target acoustic properties [7], [8]. The short duration, high frequency, broadband character of these signals make them ideal for detecting and localizing small prey such as fish and squid [1]. The dolphin auditory system has evolved to be sensitive to a broad range of frequencies as high as 150 kHz, with maximum sensitivity typically between 40–60 kHz [9]. The dolphin’s ability to echoically detect, discriminate, recognize, and locate objects can exceed human-made sonar systems, especially in noisy and reverberant environments [5]. Although laboratory studies have revealed much about how echolocation works under controlled conditions (for a review see Au, 1993), less is known about how and when dolphins echolocate in their natural environment due to the difficulty observing underwater behavior. Recent evidence demonstrates dolphins not only use echolocation to find and capture prey, but they use it to coordinate group behavior during cooperative foraging [1]. During most of the night, spinner dolphins (*Stenella longirostris*) cooperatively herd and maintain dense prey patches, in order to feed more efficiently [10]. Cooperative herding is mediated by monitoring prey and the position of group members through echolocation, which suggest dolphins can echolocate for at least most of the night [1].

Like dolphins, bats may continuously echolocate for extended periods [11]; however most seek shelter and sleep during the day. In contrast, dolphins do not appear to sleep like terrestrial mammals. Instead, unihemispheric sleep has been observed, where slow waves are seen from the cerebral cortex and thalamus on one side of the brain while the opposite side of the brain shows awake physiology [12]. One hemisphere maintains sensory awareness and motor control while the other sleeps [13], [14], [15]. Paradoxical sleep associated with hypotonia and hyporeflexia has not been observed in odontocete cetaceans [16] and would likely result in drowning. Evolutionary pressures selecting for unihemispheric slow wave sleep and the absence of paradoxical sleep, are hypothesized to include: respiratory behavioral demands [13], thermoregulation [17], and continuous vigilance [18]. Dolphins live in cooperative groups and must continuously monitor the location of group members to maintain group cohesion. Even during unihemispheric sleep, where often one eye is closed, the open eye is preferentially gazing in the direction of group members which suggest monitoring group members has a survival advantage [19]. Although predator detection and avoidance will influence vigilant behavior, direct field evidence is difficult to obtain. There is, however, evidence that bottlenose dolphin distribution is modulated by both prey abundance and predation risks, suggesting dolphins monitor their environment for predators [20].

Long-term vigilance was previously demonstrated in bottlenose dolphins in a passive hearing and visual discrimination task lasting 5 days (120 hrs) [15], [18]. These tasks required the dolphin to acoustically monitor a frequently occurring 0.5-sec tone and report when the duration changed to 1.5 sec. In addition to the acoustic tasks, the dolphins were required to periodically perform a visual discrimination tasks. A high level of performance was maintained throughout the duration of each experiment on the acoustic tasks, with a slight degradation in performance in the visual tasks. Vigilance through passive listening may be similar to a dolphin monitoring changes in whistles of other dolphins. Whistles have been hypothesized to play an important role in maintaining group cohesion [21], but recent evidence suggests echolocation is the primary means for coordinating group behavior, at least during foraging [1]. Hypothetically, echolocation should be available to the dolphin on a long-term, continuous basis in order to maintain group cohesions, and possibly avoid predators. We therefore tested the dolphin’s ability to scan its environment through echolocation and report the presence of targets for periods of 5 days and 15 days without rest.

## Materials and Methods

**Figure 1:**
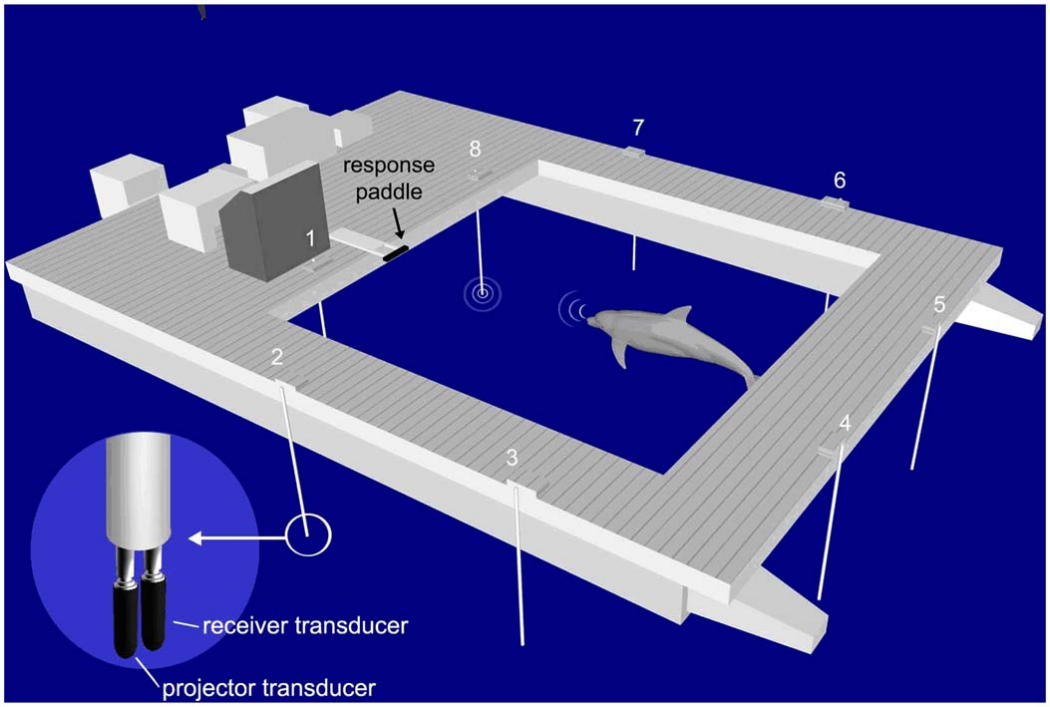
The experimental pen. Testing sessions were conducted in a mobile pen with eight transducer pairs (one a receiver and one a projector) and a response paddle. Each hydrophone was a Reson 4013. The dolphin was required to echolocate on each of the eight hydrophone locations and press the response paddle if a phantom echo was detected.

The study followed a protocol approved by the Institutional Animal Care and Use Committee of the Biosciences Division, Space and Naval Warfare Systems Center Pacific, and all applicable U.S. Department of Defense guidelines for the care of laboratory animals. Two bottlenose dolphins (*Tursiops truncatus*) participated in the study. SAY (female, age 30) participated in two previous vigilance studies [18] [15] and NAY (male, age 26) participated in one previous vigilance study [18]. All experiments were conducted in a portable floating pen (Figure 1) with netted enclosures located in San Diego Bay. The inner dimensions of the experimental pen were 6.1 m x 6.1 m while the outer dimensions were 8.4 m x 7.9 m. The pen’s net allowed sound to pass freely.

The dolphin’s task was to continuously search the perimeter of the experimental pen for phantom targets using its echolocation sense. If a phantom target was detected, the dolphin was required to press a response paddle to report the detection. Phantom targets are electronically simulated targets, generated by recording a dolphin’s echolocation pulse and convolving it with the recorded or modeled impulse response of a physical target. The resulting waveform was then amplified, delayed, and broadcast to the dolphin in real-time [22]. Like echoes from physical targets, echoes from a phantom target vary with the dolphin’s outgoing click amplitude and spectral profile as well as the range between the dolphin and the simulated target. Range was simulated by delaying and attenuating the echo. The delay between the time the dolphin emitted an echolocation click and the time the dolphin received an echo was a function of the simulated two-way travel time for sound to propagate from the dolphin, reflect off of the target, and propagate back again to the dolphin, assuming a sound speed of 1500 m/s. For a complete description of the phantom echo generator used in the experiment see Finneran et al., (2010). This approach provides the illusion that the dolphin is echolocating on an actual physical target. Previous experiments have demonstrated, that dolphins behave in a similar manner when echolocating on physical targets or phantom targets [22], [23]. Custom software randomly selected (from a rectangular distribution) a target’s range, location, and time of occurrence. Target ranges randomly varied from 98 m to 302 m in 12 m increments. Target location randomly varied from 0 to 315 degrees in 45 degree increments, and time of target occurrence randomly varied from 2 to 30 minutes in 1 second increments. In this fashion, the dolphins had no knowledge of where or when a phantom target would occur and would have to continuously echolocate on the eight stations to detect a phantom target. During a trial, target echoes generated by the phantom echo generator (PEG) would only occur at one of the eight target locations. Echolocating on the remaining seven locations did not produce phantom echoes. Targets were simulated (i.e., phantom echoes would be generated if the dolphin echolocated on the target location) for a total duration of two minutes. During this time, if a target was detected, the dolphin was required to report the detection by pressing a response paddle (Figure 1). If a correct detection resulted, the dolphin received acoustic feedback (computer generated tone that served as a secondary reinforcer), fish reinforcement and the countdown to the next trial (inter-trial-interval) began. If the dolphin reported a target but no target was present (false alarm) the dolphin received no feedback or fish reinforcement and two additional minutes were added to the countdown until the next target presentation. Late responses (i.e., responses occurring after the 2-minute response window) were considered false alarms. If a target was present but the dolphin failed to press the response paddle, a “miss” was logged, the dolphin received no feedback or fish reinforcement, and the countdown to the next trial began. Fish reinforcement was randomly varied between one and four fish and was fed by hand from a trainer. Each dolphin’s total daily consumption was spread out over each 24 h period.

Custom software, on a separate computer, continuously monitored the dolphin’s acoustic behavior at all eight hydrophone locations. Dolphin echolocation signals were converted to analogue voltages using Reson 4013 hydrophones at each of the eight hydrophone stations (See Figure 1). Analog signals were then amplified and high pass filtered at 5 kHz (Reson VP1000) which amplified the high frequency, echolocation signals but attenuated much of the ambient noise in San Diego Bay. Analogue signals were then digitized (100 kS/s) with a National Instruments PCI-MIO-16E-1 multifunction board. If the SPL_p-p_ exceeded a preset threshold level at any of the eight hydrophones, the software determined which of the eight hydrophones had the highest SPL_p-p_ and a click was logged at that location. A maximum of one click could be logged every 100 ms. The threshold level of the click detector was calibrated so only dolphin echolocation signals of the participating dolphin would exceed the threshold. Periodic sessions were conducted with no dolphin present in the pen, to ensure the ambient noise of San Diego bay, and dolphins housed in nearby pens, did not trigger false clicks. Logged clicks were saved to disk and later analyzed (Matlab 2007, The Mathworks Inc., Natick, MA, 2007). Clicks counted at each hydrophone location were added together to determine the total number of clicks the dolphin produced as a function of time. The number of clicks per minute was calculated and a 5 hr averaging window (30 minute overlap between each window) was used to smooth the click data prior to analysis.

Initial training began with a single receiver-projector pair (i.e., one location rather than eight). When the dolphin was competent at detecting and reporting phantom echoes at one station, additional stations were added, one at a time. When the dolphin was competent at detecting and reporting phantom echoes at all eight locations, the duration of the training session was increased from less than an hour, to eight hour sessions. A baseline level of performance (>80% correct) during an eight hour session was required before the 5-day testing sessions were conducted. Each dolphin participated in three, 5-day testing sessions. The 5-day sessions were conducted in San Diego Bay near the pen complex where the dolphins were housed. The testing pen was separated from the main pen complex by 12 m. SAY was chosen based on her superior performance to participate in an additional 15-day testing session. The 15-day session was conducted at an isolated location in San Diego Bay using the same, mobile, experimental pen. On non-testing days, the dolphins participated in a variety of tasks including maintenance of behaviors related and unrelated to the study. Training on non-testing days typically occurred between the hours of 0600 and 1600.

Data were analyzed using the lme4 package [24] designed for mixed effect modeling in R [25]. Because the responses of each dolphin were measured over time (repeated measures design), dolphin *subjects* (SAY and NAY) were modeled as random effects while *session* number (1–3), *day* number (1–5) and *diel* pattern (day or night) were modeled as fixed effects. Outcome variables included *misses*, *false alarms (FA)*, and *response latency (RL)*. Models were simplified by fitting a maximum model and then removing non-significant terms starting with higher-order interactions. Normality and homogeneity were visually inspected by plotting residuals against fitted values. To test the validity of each mixed effects model, likelihood ratio tests [26] were conducted between simplified models (containing both random fixed effects and fixed effects), and null models (containing only random effects).

## Results

**Figure 2:**
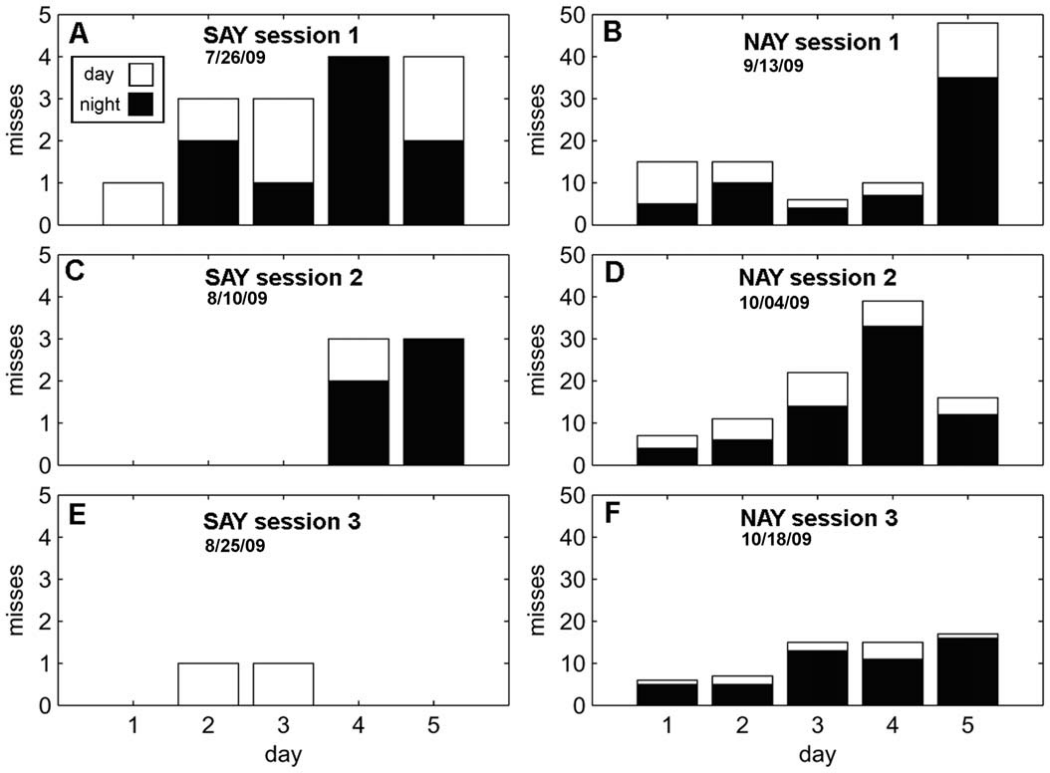
Number of *misses* for 5-day testing sessions. The left column (A,C,E) are plots from SAY’s three sessions while the right column (B,D,F) are plots from NAY’s three sessions. The number of *misses* during the day (white) is stacked on top of night *misses* (black). Values represent cumulative misses for each day.

A summary of the results from the 5-day sessions is in Table 1. Both dolphins were able to continuously echolocate and report the presence of phantom targets for five days with a high degree of accuracy. SAY demonstrated superior performance with only two misses after continuously echolocating for 5 days (Figure 2E). Table 2 presents parameters from the best-fit mixed effects models for each outcome variable.

**Table 1:** Performance for 5-day sessions.

	SAY			NAY		
Start date	26 JUL	10 AUG	25 AUG	13 SEP	04 OCT	18 OCT
**Correct detections** **(%)**	96.3	98.6	99.6	75.3	77.9	86.2
**Total trials**	403	418	454	421	430	434
**Misses (total)**	15	6	2	104	95	60
**False alarms (total)**	7	0	5	15	13	11

**Table 2:** Model parameters for the outcome variables *miss, false alarms*, and *response latency*.

Miss		False Alarm		Response Latency	
*p<0.001*		*p<0.001*		*p<0.001*	
intercept = 0.692		intercept = −0.725		intercept = 32.780	
day	b = 1.92	day	b = 0.34	day	b = 3.17
ses*day*die	b = −0.64	day*die	b = 0.36	ses*day	b = −0.79
				ses*day*die	b = −0.32

Likelihood ratio tests (p-values) were conducted between mixed-effects models and their corresponding null models to select the simplest explanatory model for each outcome variable (see text for more detains). Only significant fixed effects and interaction are presented. ses = session, die = diel.

**Figure 3:**
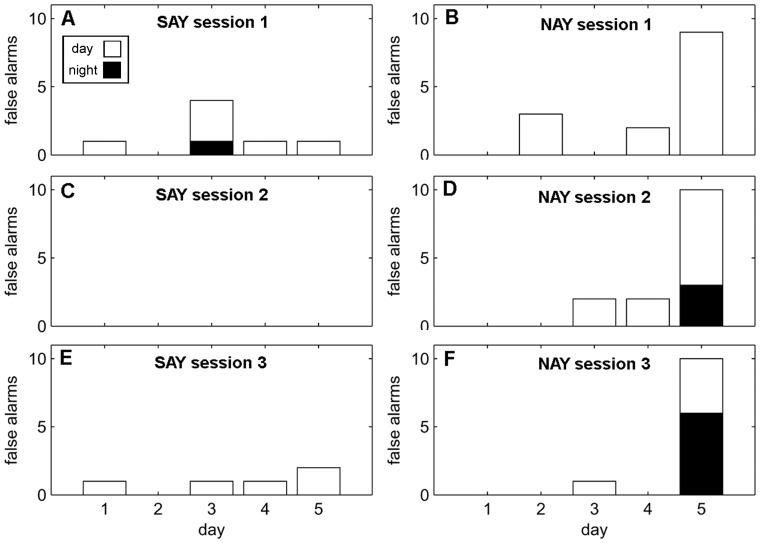
False alarms for 5-day testing sessions. The left column (A,C,E) are plots from SAY’s three sessions while the right column (B,D,F) are plots from NAY’s three sessions. The number of *false alarms* during the day (white) is stacked on top of night *false alarms* (black). Values represent cumulative False alarms for each day.

**Figure 4:**
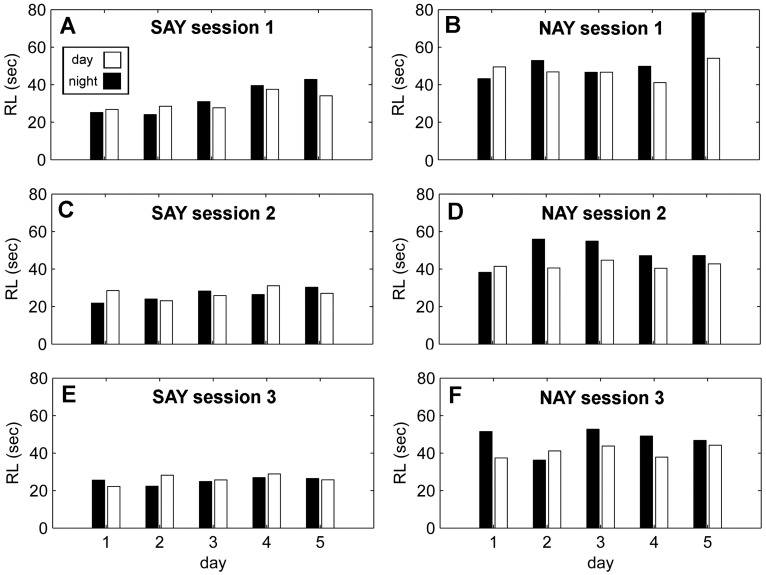
Response latency (RL) for 5-day testing sessions. The left column (A,C,E) are plots from SAY’s three sessions while the right column (B,D,F) are plots from NAY’s three sessions. The average RL during the day (white) is adjacent to the average RL during the night (black).

**Figure 5:**
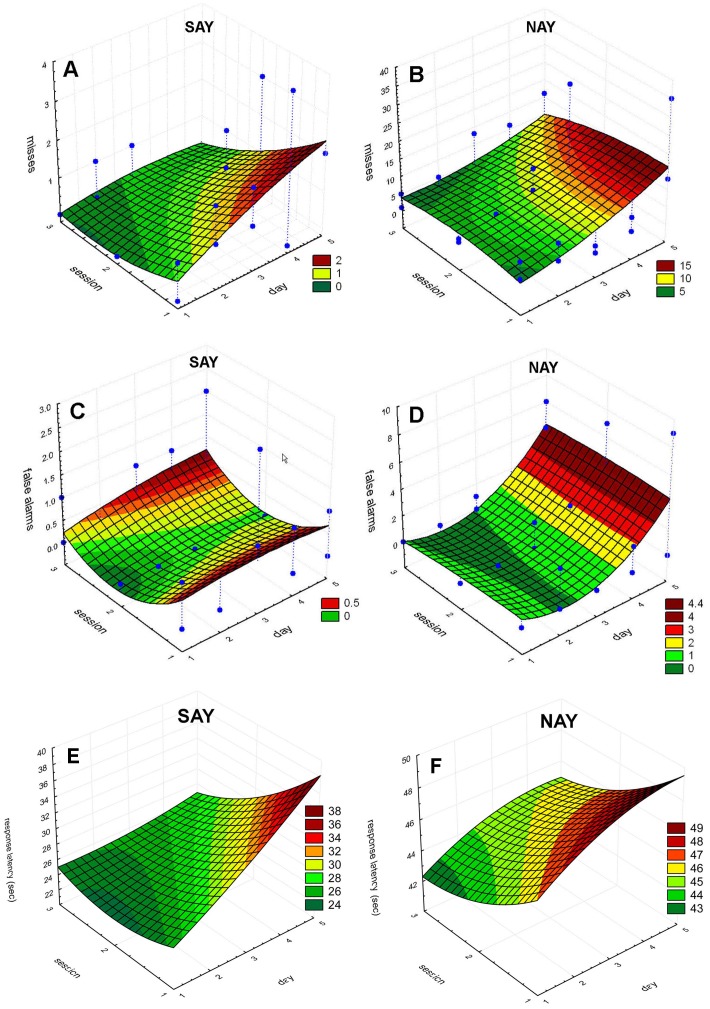
Surface plots describing dolphin performance for 5-day sessions. Data and surface plots were generated for misses (A,B), false alarms (C,D) and response latency (E,F) as a function of session number and day number. Surfaces were estimated using a quadratic polynomial fit (Statistica 7.1, StatSoft, Inc. 1984–2005). Data points are not plotted for response latency to preserve clarity of the figure.

A significant main effect of *day* and a significant three-way interaction between *sessions, day, and diel*, suggest the pattern of *misses* can best be characterized by an increase in *misses* over each five day period (Figure 2). However, the total number of *misses* decreases each *session*, and are more likely to occur at night (Figure 2). A significant main effect of *day* and a significant interaction between *day* and *diel* best characterize the pattern of false alarms. There was a tendency for false alarms to increase over the duration of each 5-day session (Figure 3). Most of the false alarms occur during the day towards the beginning of each session, but shift towards night by the end of a 5-day session. False alarm rate did not significantly change (p>0.05) over sessions (Table 1). The best-fit model characterizing response latency is complex with a significant main effect of *day*, a significant two-way interaction between *session* and *day* and a significant three-way interaction between *session, day*, and *diel* (Figure 4). The interaction between session and day can be clearly seen in Figure 5E and Figure 5F. Response latency increased during the initial 5-day session which suggests the dolphins were becoming fatigued or unmotivated. However, the size of this effect diminished over the three sessions.

**Figure 6:**
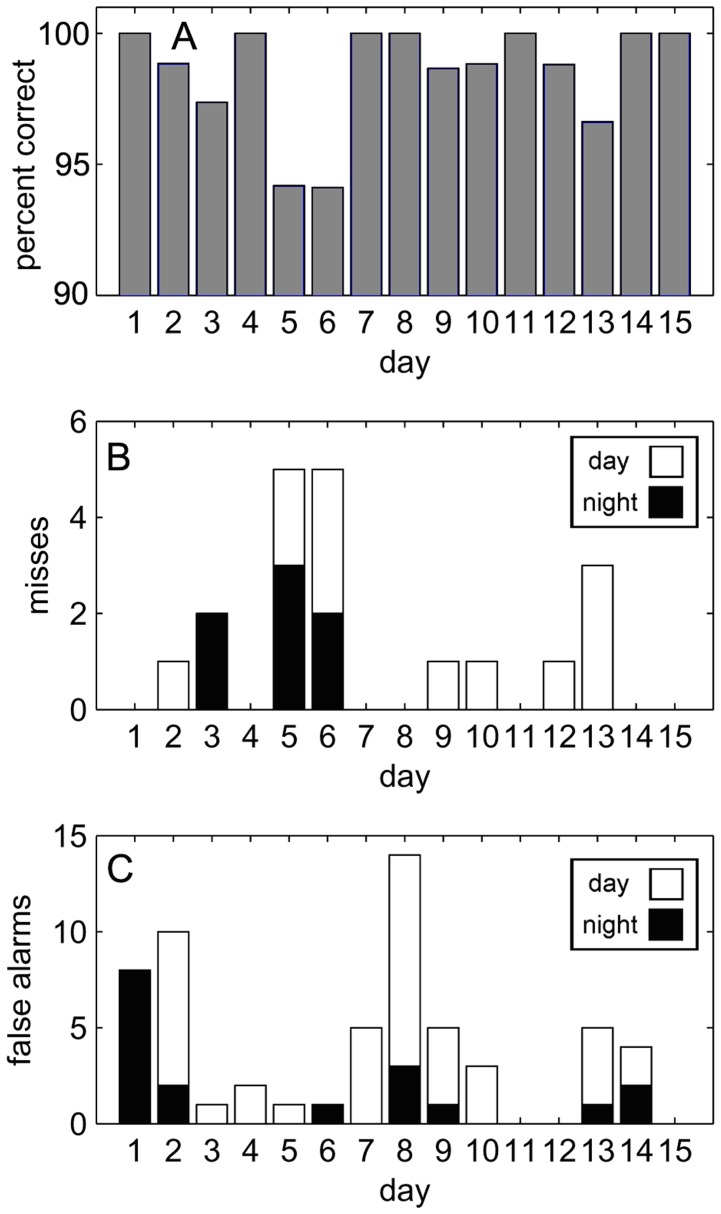
Percent correct for the fifteen day session. SAY was near ceiling level performance for the duration of the session (A). The average number of trials per 24 hr period was 78.4. Diel patterns (stacked bar graph) for *misses* (B) and *false alarms* (C) during the fifteen day session. There were no significant trends in SAY’s performance.

A planned 30-day vigilance session began on 6 January 2010, with dolphin SAY. On 20 January 2010 (day 15) the experiment was terminated due to the onset of a winter storm. Except for the extended duration of the experiment and the location of testing pen, the methodology was identical to the 5-day testing sessions. Figure 6 illustrates percent correct over the 15-day experiment. There was an average of 78.4 (STD = 15.2) trials per 24 hr period. SAY demonstrated a remarkable ability to continuously perform the tasks for 15 days. Dolphin performance was modeled using the same mixed effects procedure as the 5-day sessions, except *session* was not a predictor since only one session was conducted. There were no significant main effects or interactions (null hypothesis could not be rejected) for any of the outcome variables (*miss, FA, RL*).

**Figure 7:**
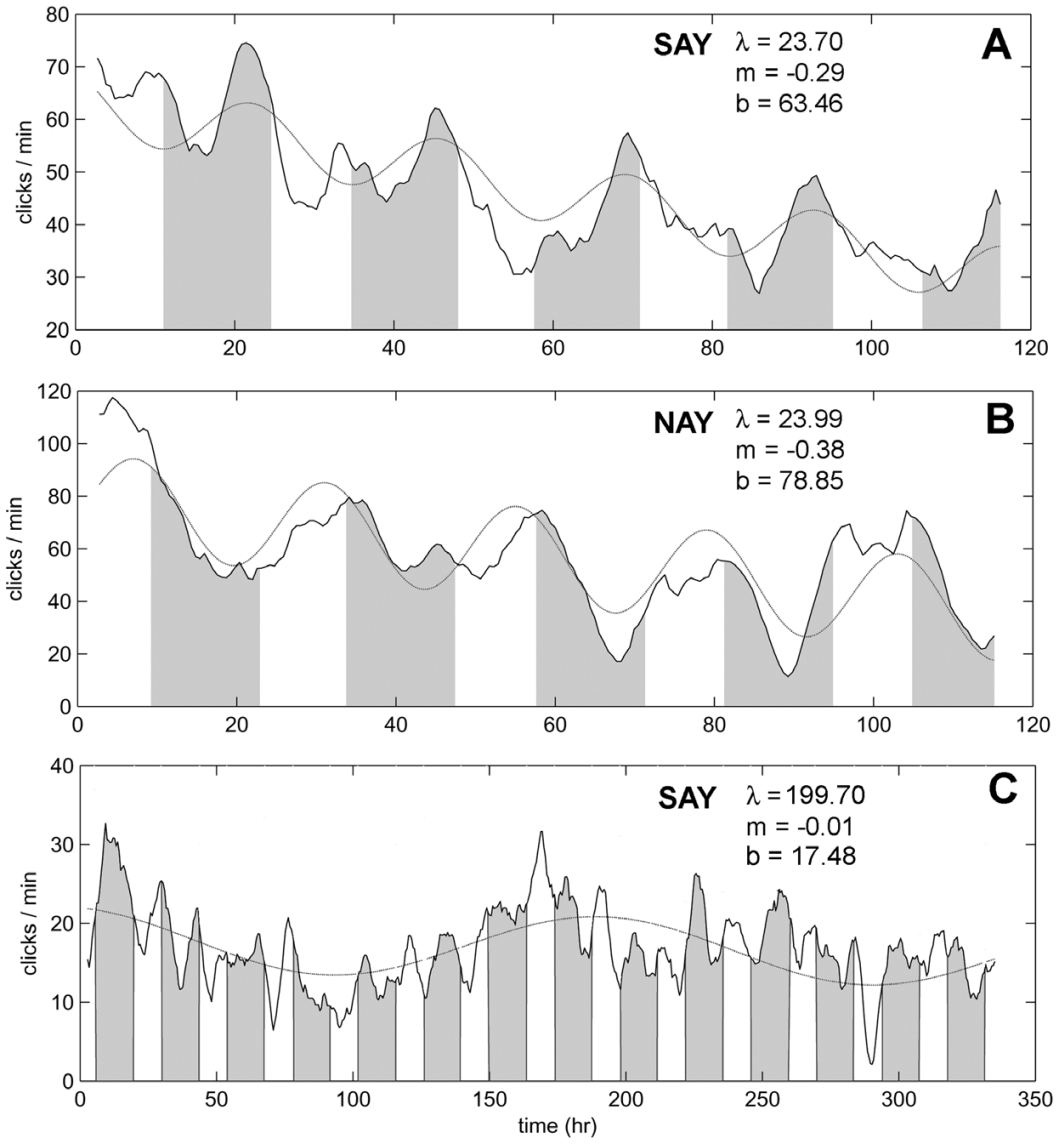
The number of clicks/minute as a function of elapsed time in hours (thick line). (A) and (B) show data from the last 5-day session each dolphin participated in and (C) is data from the 15-day session. Model predictions are plotted as thin lines with their respective parameters. Shaded areas represent night while unshaded areas represent day. Both dolphins have a diel pattern with a period (λ) of about 24 hrs and a negative slope (b) for their last 5-day session. The model was not appropriate for the 15-day session.

The echolocation behavior of both dolphins from their last 5-day training session is plotted in Figure 7A and Figure 7B. SAY’s 15-day echolocation behavior is plotted in Figure 7C. Shaded areas represent night (i.e., the time between sunset and sunrise). A visual analysis of the dolphin’s 5-day echolocation behavior displays a clear cyclic pattern. However, peaks and troughs in activity differed between the dolphins. SAY was most active in the hours just before dawn while NAY was most active in the hours around sunset. Despite peak activity being out of phase, their acoustic behavior can be described by a common non-linear model consisting of a sinusoid modulated by a decreasing slope:
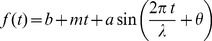
(1)where *f(t)* is a function representing clicks per minute, *b* and *m* are the *y* intercept and slope of the linear component respectively, *t* is units of time, and *a*, *λ*, and *θ* are the amplitude, period, and phase of the sinusoidal component. Model parameters were estimated using the nlme package [27] designed for non-linear mixed effect modeling in R [25]. Best-fit model parameters are displayed in Table 3. For the 5-day sessions, both dolphins displayed a decrease in the number of clicks per minute represented by negative slopes (m). Diel patterns with a period (λ) very close to 24 hrs were apparent with both dolphins and the diel amplitude (a) was largest with for dolphin NAY. Model fits for the 15-day session had a slope (m) close to zero with a period (*λ*) close to 200 hrs.

**Table 3:** Statistics and model parameters for the dolphins’ acoustic behavior (see equation 1).

	SE	mean	*m*	*b*	*a*	*λ*	*θ*
**SAY(5day)**	9.71	46.38	−0.29	63.46	6.49	23.70	154
**NAY(5day)**		56.69	−0.38	78.85	17.78	23.99	50
**SAY(15day)**	4.20	16.96	0.01	17.48	4.20	199.7	179

SE is residual standard error of the mixed-effect model. During the 5-day session, both dolphins demonstrate a 24 hr diel cycle (λ) and a negative slope (*m*).

## Discussion

This study demonstrates that dolphins can continuously echolocate and report the presence of targets for at least several days with a remarkable degree of accuracy. During the 5-day test sessions, both dolphins demonstrated a decrease in performance within each session reflected by an increase in misses, false alarms and response latency. The cause is likely related to increased fatigue or loss of motivation. Performance decrements were less apparent by their third testing session which suggests the dolphins were learning. Exactly what they were learning remains speculative. For example, the dolphins could have become more efficient at the task, they could have been more motivated, or their long-term attention could have been enhanced. Both dolphins participated in 5-day vigilance session in previous studies [15], [18]. However, doing a task for 5 days without interruption was not part of their everyday routine. Adjusting from a 24-hour cycle, largely influenced by the work hours of their human caregivers, to a task that requires continuous performance may require an acclimation period.

Both dolphins exhibited a diel pattern in their click production with slightly long-term negative slopes, for the 5-day sessions. The non-linear model (equation 1) was chosen primarily for parsimonious reasons. The model consisted of only one, modulated sinusoid. More complex models (e.g., with multiple sinusoids) might better describe the diel pattern seen with SAY in Figure 7A. A secondary peak with a period of approximately 12 hours is visually noticeable which hints at crepuscular activity. Diel patterns are also found in some dolphin populations [28]. For example, Hawaii spinner dolphins (*Stenella longirostris*) feeding and resting behavior is governed by the diel migration pattern of the mesopelagic prey they feed upon. Curiously, diel patterns were non-coherent between the dolphins in the current study, SAY being most active before sunrise while NAY was most active before sunset. Both dolphins were subjected to the same experimental procedures, had the same feeding schedules, and were housed in the same area. Without further testing, the variables responsible for the diel echolocation patterns cannot be identified with confidence.

There were large individual differences in performance between the dolphins, with SAY the female typically outperforming the male NAY. The source of this difference may be related to experience, since SAY had participated in two previous long-term vigilance studies [15], [18] while NAY only participated in one [18]. However, the subjective opinion of the authors is that individual differences were “personality” related. Unlike NAY, SAY appeared to be highly motivated and eager to participate in this study, often producing victory squeals [29] when correctly responding to a positive target. Because of her superior performance, SAY was selected for the 15-day experiment.

During the 15-day session, SAY displayed no indication of deteriorating performance. How much longer she could have performed the task is unknown. The pattern of SAY’s click production during the 15-day session was less structured and a simple sinusoidal model with a 200 hr period lacks a suitable a priori hypothesis. Despite obvious peaks and valleys in her click production, peak click production occurred at night or day without a consistent pattern, even during the first 5 days. The slope of the linear component of the model (m) was negligible, indicating the number of clicks over a longer time scale was stable and not in decline. SAY produced almost one third as many clicks during the 15-day session compared to her last 5-day session but maintained a very high level of performance. It appears that SAY learned to be more efficient (i.e., detect phantom targets using less clicks) in this task, but it must be noted that the 15-day experiment took place at a different location than the 5-day sessions. Differences between the testing sites, such as background noise and proximity to other dolphins, may have been factors.

Unihemispheric sleep is thought to have evolved in cetaceans (and some pinnipeds) to facilitate breathing at the surface [13]. Although respiratory demands may be the primary factor selecting for continuous swimming and awareness, unihemispheric sleep may have a secondary function, to aid in long-term vigilance. Unihemispheric sleep is also found widespread in birds and is used for predator detection. Birds sleeping in groups display a greater tendency for unihemispheric sleep when they were located at the outer edge of the group where predation risk is greatest [30]. Furthermore, birds sleep with one eye open, facing away from the group where predators are most likely detected. Dolphins sleep with one eye open as well [31]. One study found that the open eye of resting dolphins was oriented towards group members more often than away from the group where predation risk is greatest [19]. However, this study was conducted in a tank where there was no risk of predation. In this case, the benefit of monitoring group members was greater than monitoring for non-existent predation. If vision is restricted during the night or in murky waters, echolocation will be the primary means for monitoring the environment, for either group members or predators. Many dolphin populations are exposed to an almost constant risk of shark attack [32]. Although direct observations of attacks on dolphins are difficult to observe, many dolphins have bite marks that can be used to estimate shark attack prevalence. One study measured bite marks on 74% of the non-calve bottlenose dolphin population in western Australia [33]. Not surprisingly, dolphins selectively choose habitats where shark density is lowest or where sharks are easier to detect [20]. Dolphins also increase their group size when feeding in areas of higher shark density. One reason a larger group will aid in protection is that the effectiveness of detecting a shark increases with group size [34] due to shared vigilance. The majority of shark attack victims are young dolphins [35] which explains why bottlenose dolphin mothers with neonates have also been observed continuously swimming (a vigilant state) for at least two months postpartum [36]. In this case, mother and calf likely maintain heightened awareness due to calf vulnerability to predation, and to prevent the calf from getting separated from its only food source (its mother’s milk). Mothers in the wild will have to monitor their calf and group members and if vision is restricted, echolocation will be the primary sensory modality.

Although unihemispheric sleep is hypothesized to facilitate continuous vigilance in the current study, conclusive physiological evidence is lacking. This hypothesis should be tested with a similar experiment in conjunction with electrophysiological recordings. From an anthropomorphic viewpoint, the ability of the dolphin to continuously monitor its environment for days without interruption seems extreme. However, the biological, sensory and cognitive ecology of these animals is relatively unique and demanding. If dolphins sleep like terrestrial animals, they might drown. If dolphins fail to maintain vigilance, they become susceptible to predation. As a result, the apparent “extreme” capabilities these animals possess are likely to be quite normal, unspectacular, and necessary for survival from the dolphin’s perspective. Although much can be gained by observing dolphins in the field, complimentary laboratory studies are necessary to document the full range of these animals’ capabilities.
